# Prevalence and correlates of diabetes mellitus in Malawi: population-based national NCD STEPS survey

**DOI:** 10.1186/1472-6823-14-41

**Published:** 2014-05-12

**Authors:** Kelias Phiri Msyamboza, Chimwemwe J Mvula, Damson Kathyola

**Affiliations:** 1 World Health Organisation, Malawi Country Office, City Centre, P.O. Box 30390, Lilongwe 3, Malawi; 2 University of Malawi, College of Medicine, Community Health Department, Lilongwe, Malawi; 3 Ministry of Health, Lilongwe, Malawi

**Keywords:** Diabetes, Non-communicable diseases, Sub-Saharan Africa, Malawi

## Abstract

**Background:**

Previously considered as a disease of the *affluent, west or urban* people and not of public health importance, diabetes mellitus is increasingly becoming a significant cause of morbidity and mortality in sub-Saharan Africa. However, population-based data to inform prevention, treatment and control are lacking.

**Methods:**

Using the WHO STEPwise approach to chronic disease risk factor surveillance, a population-based, nationwide cross-sectional survey was conducted between July and September 2009 on participants aged 25–64 years. A multi-stage cluster sample design and weighting were used to produce a national representative data for that age range. Detailed findings on the magnitude of diabetes mellitus and impaired fasting blood glucose are presented in this paper.

**Results:**

Fasting blood glucose measurement was conducted on 3056 participants (70.2% females, 87.9% from rural areas). The age- sex standardised population-based mean fasting blood glucose was 4.3 mmol/L (95% CI 4.1-4.4 mmol/L) with no significant differences by age, sex and location (urban/rural). The overall prevalence of impaired fasting blood glucose was 4.2% (95% CI 3.0%-5.4%). Prevalence of impaired blood glucose was higher in men than in women, 5.7% (95% CI 3.9%-7.5%) vs 2.7% (95% CI 1.6%- 3.8%), *p* < 0.01. In both men and women, prevalence of raised fasting blood glucose or currently on medication for diabetes was 5.6% (95% CI 2.6%- 8.5%). Although the prevalence of diabetes was higher in men than women, 6.5% (95% CI 2.6%-10.3%) vs 4.7% (95% CI 2.4%-7.0%), in rural than urban, 5.4% (95% CI 2.4%-8.4%) vs 4.4% (95% CI 2.8%-5.9%) and in males in rural than males in urban, 6.9% (95% CI 2.8%-11.0%) vs 3.2% (95% CI 0.1%-6.3%), the differences were not statistically significant, *p* > 0.05. Compared to previous estimates, prevalence of diabetes increased from <1.0% in 1960s to 5.6% in 2009 (this study).

**Conclusion:**

High prevalence of impaired fasting blood glucose and diabetes mellitus call for the implementation of primary healthcare approaches such as the WHO package for essential non-communicable diseases to promote healthy lifestyles, early detection, treatment and control.

## Background

Diabetes mellitus alongside other non-communicable diseases (NCDs), mainly cardiovascular diseases, cancers and chronic respiratory diseases are increasingly becoming significant public health problems globally. In 2010, the world prevalence of diabetes among adults (aged 20–79 years) was estimated at 6.4%, affecting 285 million adults, and is projected to increase to 7.7%, and 439 to 552 million adults by 2030. Between 2010 and 2030, there will be a 69% increase in numbers of adults with diabetes in developing countries and a 20% increase in developed countries [1,2].

In sub-Saharan Africa, it was estimated that 12.1 million people were living with diabetes in 2010 and this was projected to increase to 23.9 million by 2030. Type 2 diabetes is the most common form accounting for over 90% of the cases. Type 2 diabetes is becoming more prevalent owing to rising rates of obesity, physical inactivity, urbanisation and nutritional transitions [3-5]. Due to barriers in accessing diagnosis and treatment including lack of diagnostic tools, glucose monitoring equipment and high cost of diabetes treatment; diabetic patients are often unaware, undiagnosed and those on treatment are poorly managed and monitored with glucose levels adequately controlled in less than a third (27%) and 60% or more of the patients have complications [3,6].

In Malawi, studies conducted in 1960s and 1970s demonstrated that diabetes was not an important public health problem then with prevalence of 1% or less [7,8]. Under-nutrition was a major public problem where one in three (36%) adults was undernourished while overweight (body mass index ≥25 Kg/m^2^) was less than 7% [9]. However, epidemiological transition has occurred just like in other countries in sub-Saharan Africa. Overweight and obesity, not under-nutrition, is now a major public health problem in adults where up to one in four (28%) females is overweight [10]. However, detailed population-based data on the prevalence and correlates of diabetes to inform baseline data for surveillance, policies and interventions are scarce. Between July and September 2009, we conducted a nationwide population-based cross-sectional survey using WHO STEPS survey tools to determine the magnitude of chronic non-communicable diseases and their risk factors in Malawi. Age-sex-standardised prevalence of diabetes was 6.5%, 4.7%, 5.6% in males, females and both males and females respectively [11]. In this paper, the detailed findings on the population-based prevalence and correlates of diabetes in Malawi are presented.

## Methods

### Study design and sample size

This study was a nationwide population- household- based cross-sectional survey designed according to a WHO STEPwise approach to chronic disease risk factor surveillance [12]. Sample size, calculated using the standard formula, was adjusted for design effect for complex sample design set at 1.5, age-sex estimates in 25–64 age range (8, 10-year intervals) and a non-response rate of 20%. With these adjustments, the final required sample size was 5,760. It was assumed that the non-response rate would be high because participants may refuse blood testing and/ or not adhere to fasting, the latter being required for fasting blood glucose testing.

### Sampling of survey sites, households and eligible participants

Enumeration areas (EAs) were used as survey sites. Administratively, Malawi is divided into twenty-eight districts. In turn, each district is sub-divided into smaller administrative units called traditional authorities (TAs). Each TA is sub-divided into EAs by the National Statistical Office (NSO). Enumeration areas are classified as urban or rural. Each EA has demographic data and a sketch map. The sketch map shows the EA boundaries, location of buildings, and other landmarks. The list of all EAs in Malawi from latest (2008) population and housing census was obtained from NSO. This list was used as a sampling frame for the random selection of EAs. According to the WHO NCD STEPS Survey manual Part 2 Study design and sample size, in each EA, 30–50 households could be selected and in each household only one eligible participant could be selected [12]. We settled for 40 households per EA. Therefore to reach the required sample size, the total number of EAs to be selected was 144 EAs (5,760/40). The 144 EAs were randomly selected nationwide using the probability proportional to size (PPS) sampling method. In each EA, 40 households were randomly selected using systematic sampling method. Sampling interval was calculated by dividing the total number of households in the EA as given by the NSO by 40 (the number of households to be selected). At household level, only one eligible participant was selected using the Kish sampling method, built-in personal digital assistant (PDA, HP iPAQ). Households with no eligible participant were not replaced.

### Recruitment of participants and data collection

Eligible participants were all adults aged 25–64 years. Participants were involved in the study for two days. Day one was for the questionnaire and anthropometric measurements. Day two was for blood pressure measurement and laboratory tests. Formal written consent was obtained. Participants with abnormal physical or laboratory findings as defined below were counselled and referred to their nearest health facility for further action and follow up. Body measurements and laboratory tests were performed by nurses and clinical officers while enumerators conducted the interviews. A total of seven survey teams, each with 8 members were deployed to collect data over a period of 30 days between July and September 2009.

### Demographic and lifestyle data collection

Demographic and lifestyle data were collected using WHO STEPS questionnaire. The questionnaire was programmed on PDA. It consisted of core (age, sex and education in years and current exposure to tobacco and alcohol, diet and physical activity), expanded (rural/urban setting, occupation, average household income) and optional (marital status, medical and health history, past history of smoking and alcohol consumption) variables. The medical and health history component included questions on medication, cigarette use, diabetes mellitus and hypertension. The English questionnaire was translated into two main local languages (*Chichewa* and *Tumbuka*).

### Biochemistry laboratory measurements

On the first day of the survey participants were asked to starve overnight. Consenting participants were asked not to consume any food except for water after taking supper/dinner of that day until the survey team came again in morning of the following day (day 2). People converged at the agreed place in their community where finger prick blood samples were taken. Those that complied with the advice (starving overnight) were eligible for finger prick blood sample collection. Fasting blood glucose was measured using Accutrend® *Plus* machines (Roche, Mannheim, Germany).

### Data management

Data were collected electronically using PDAs programmed with WHO e-STEPS software. Data on the PDAs were downloaded into the computer installed with WHO NCD STEPS software. The files of each participant were then merged using the participant identity (PID) number cross-checked with participant name, EA number or village/township name and other particulars where necessary. After merging, common variables in the dataset were matched and inconsistencies were corrected.

Data were weighted by calculating sample weights for all records using the probability of selection at each stage of sampling. Thus, for each participant his/her weight was calculated by multiplying the probability of EA selection, the probability of household selection, the probability of participant selection within the household and age-sex population distribution in Malawi. The participant’s weight was equal to the inverse of this product. Data analysis was conducted using WHO e-STEPs software and Epi Info, version 3.5.1 (Centres for Disease Control and Prevention, Atlanta, Ga). Chi-square tests were used to evaluate differences in proportions and student’s t-test for differences in means.

### Definitions

Impaired blood glucose was defined as fasting capillary whole blood value of ≥5.6 mmol/L (100 mg/dl) but less than 6.1 mmol/L (110 mg/dl). Diabetes was defined as fasting capillary whole blood glucose level >6.1 mmol/L (>110 mg/dl) or currently on medication for diabetes mellitus (documented in the health booklet). Results were considered statistically significant, *p* < 0.05 or *t* < 0.05.

### Ethics statement

Ethical approval was granted by the Malawi National Health Sciences Research and Ethics Committee. Written informed consent was obtained before participants were enrolled in the study using the WHO NCD STEPS survey consent form.

## Results

### Socio-demographic characteristics of participants enrolled in the study

A total of 5451 eligible adults were selected and approached to participate in the survey. Of these, 245 (4.5%) refused/were not available while 5206 (95.5%) consented and took part in the survey. Of the 5206 participants that took part in the survey, 183 (3.5%) were pregnant women and were excluded in the analysis for this paper to control for the potential influence of pregnancy on blood glucose levels. Complete data for fasting blood glucose measurement were available and analysed for 3056 (60.8%) of the 5023 eligible non-pregnant participants. Of the 3056 participants with complete fasting blood glucose data, 2145 (70.2% were females, 2050 (67.1%) were aged 25–44 years, 2685 (87.9%) were from rural areas, 2619 (85.7%) had primary education or none, 2234 (73.1%) were married or cohabiting, 364 (10.9%) were tobacco smokers, 403 (13.2%) were alcohol drinkers and 669 (21.9%) were overweight or obese (body mass index 25.0 Kg/m^2^ or more). Table 1 summarises the socio-demographic characteristics of participants enrolled in the study.

**Table 1:** Characteristics of participants enrolled in Malawi NCD STEPS survey 2009

	**Total**		**Male**			**Female**		
	**n**	**%**	**n**	**%**	**95% CI**	**n**	**%**	**95% CI**
**Gender:**	5,206	100	1,690	32.5	30.3-34.7	3,516	67.5	66.0-69.1
**Age (years):**								
25-34	2,335	44.9	719	42.5	38.9-46.1	1,616	46.0	43.6-48.4
35-44	1,321	25.4	459	27.2	23.1-31.3	862	24.5	21.6-27.4
45-54	902	17.2	296	17.5	13.2-21.8	604	17.2	14.2-20.2
55-64	650	12.5	216	12.8	8.3-17.3	434	12.3	9.2-15.4
25-64	5,206	100.0	1,690	100.0	-	3,516	100.0	-
**Marital status:**								
Never married	161	3.1	91	5.4	0.8-10.0	70	2.0	-1.3-5.3
Currently married	3,819	73.5	1,475	87.4	85.7-89.1	2,344	66.8	64.9-68.7
Separated/divorced	754	14.5	99	5.9	1.3-10.5	655	18.6	15.6-21.6
Widowed	464	8.9	22	1.2	-3.4-5.8	442	12.6	9.5-15.7
Total	5,198	100.0	1,687	100.0	-	3,511	100.0	-
**Education:**								
None	1,285	24.7	237	14.0	-	1,048	29.8	-
Standard 1-5	1,807	34.8	558	33.0	-	1,249	35.6	-
Standard 6-8	1,391	26.7	539	31.9	-	852	24.2	-
Secondary and above	720	13.8	355	21.1	-	365	10.4	-
Total	5,203	100.0	1,689	100.0	-	3,514	100.0	-

Key: n = number in the group, % = percentage, CI = confidence interval.

### Population- based mean fasting blood glucose

The age- sex standardised population-based mean fasting blood glucose for 3056 participants was 4.3 mmol/L (95% CI 4.1-4.4 mmol/L). There were no significant differences in mean fasting blood glucose by location (urban/rural), age and sex (Table 2).

**Table 2:** Population based mean fasting blood glucose (mmol/L): Malawi NCD STEPS survey 2009

	**Men**			**Women**			**Both sexes**		
	**n**	**Mean (mmol/L)**	**95% CI**	**n**	**Mean (mmol/L)**	**95% CI**	**n**	**Mean (mmol/L)**	**95% CI**
Age group (years):									
25-34	349	4.3	4.1-4.5	897	4.2	4.1-4.3	1246	4.2	4.1-4.4
35-44	257	4.4	4.2-4.7	544	4.2	4.0-4.4	801	4.3	4.1-4.5
45-54	169	4.1	3.9-4.3	400	4.3	4.1-4.4	569	4.2	4.0-4.4
55-64	134	4.6	4.1-5.0	303	4.4	4.2-4.5	437	4.4	4.2-4.7
25-64	909	4.3	4.2-4.5	2144	4.2	4.1-4.3	3053	4.3	4.1-4.4
Residence:									
Urban	81	4.2	4.0-4.5	288	4.3	4.1-4.5	369	4.3	4.1-4.5
Rural	828	4.3	4.2-4.5	1856	4.2	4.1-4.3	2684	4.3	4.1-4.4

### Population-based prevalence of impaired fasting glucose

The overall age-sex standardised population-based prevalence of impaired fasting blood glucose (capillary whole blood value 5.6 mmol/L- 6.1 mmol/L) in 3056 participants was 4.2% (95% CI 3.0%-5.4%). Prevalence of impaired blood glucose was higher in men than women, 5.7% (95% CI 3.9%-7.5%) vs 2.7% (95% CI 1.6%- 3.8%), *p* < 0.01 (Table 3).

**Table 3:** Population based prevalence of Impaired Fasting glucose: Malawi NCD STEPS survey 2009

	**Men**			**Women**			**Both sexes**		
	**n**	**%**	**95% CI**	**n**	**%**	**95% CI**	**n**	**%**	**95% CI**
Age group (years):									
25-34	351	5.4	2.8-8.0	898	2.0	0.9-3.0	1249	3.7	2.1-5.3
35-44	257	6.1	3.2-8.9	544	1.8	0.4-3.3	801	3.9	2.3-5.5
45-54	169	5.3	2.0-8.7	400	4.1	1.9-6.3	569	4.7	2.7-6.6
55-64	134	6.7	2.4-11.0	303	5.4	2.6-8.2	437	6.0	3.2-8.7
**25-64**	**911**	**5.7**	**3.9-7.5**	**2145**	**2.7**	**1.6-3.8**	**3056**	**4.2**	**3.0-5.4**

Impaired fasting glycaemia was defined as capillary whole blood value: ≥5.6 mmol/L (100 mg/dl) but less than 6.1 mmol/L (110 mg/dl).

### Population-based prevalence of raised fasting blood glucose or currently on medication for diabetes

In both men and women, prevalence of raised fasting blood glucose (capillary whole blood value ≥6.1 mmol/L) or currently on medication for diabetes in 3056 participants was 5.6% (95% CI 2.6%- 8.5%). Although the prevalence was higher in men than women, 6.5% (95% CI 2.6-10.3%) vs 4.7% (95% CI 2.4%-7.0%), in rural than urban, 5.4% (95% CI 2.4%-8.4%) vs 4.4% (95% CI 2.8%-5.9%) and in males in rural than males in urban, 6.9% (95% CI 2.8%-11.0%) vs 3.2% (95% CI 0.1%-6.3%), the differences were not statistically significant, *p* > 0.05 (Table 4).

**Table 4:** Population based prevalence of raised fasting blood glucose or currently on medication for diabetes

	**Men**			**Women**			**Both sexes**		
	**n**	**%**	**95% CI**	**n**	**%**	**95% CI**	**n**	**%**	**95% CI**
Age group (years):									
25-34	351	6.2	1.5-10.9	898	3.6	1.8-5.4	1249	4.9	1.8-8.0
35-44	257	7.8	2.8-12.7	544	4.7	1.6-7.8	801	6.2	2.4-10.0
45-54	169	4.7	0.9-8.4	400	6.4	2.2-10.6	569	5.6	2.0-9.2
55-64	134	7.6	2.7-12.5	303	6.1	3.3-8.9	437	6.8	3.8-9.8
25-64	911	6.5	2.6-10.3	2145	4.7	2.4-7.0	3056	5.6	2.6-8.5
Residence:									
Urban	82	3.2	0.1-6.3	289	4.7	2.6-6.8	371	4.4	2.8-5.9
Rural	829	6.9	2.8-11.0	1856	4.8	2.1-7.4	2685	5.4	2.4-8.4

Raised blood glucose is defined as capillary whole blood value: ≥ 6.1 mmol/L (110 mg/dl).

## Discussion

This study is one of the few large national population-based studies that have provided latest evidence on the status of diabetes mellitus in east and southern Africa region in general, Malawi in particular. In 1960s, diabetes was not an important public health problem in Malawi and the prevalence, then, was less than 1%. Malnutrition, even in adults was the main problem where up one in three adults (36%) were under-nourished or underweight (body mass index <18.5 Kg/m^2^) [7-9]. However, this study, in agreement with other studies, demonstrated that epidemiological nutritional transition occurred in Malawi just like in other countries in east and southern Africa. Overweight and/ or obesity, not under-nutrition, is now a major public health problem in adults where up to one in five (21.9%) adults are overweight [10] and prevalence of diabetes increased from less than 1% as previously reported in 1960s to 5.6% in 2009 (this study).

This study also demonstrated that in Malawi, diabetes was just as common in rural as in urban areas and in men as in women. Apparently it was higher in rural than urban areas, 5.4% (95% CI 2.4%-8.4%) vs 4.4% (95% CI 2.8-5.9%), in men than in women, 6.5% (95%CI 2.6%-10.3%) vs 4.7% (95% CI 2.7%-7.0%) but the differences were not statistically significant (*p* >0.05). Number of participants that were tested for fasting blood glucose was relatively smaller in urban than rural (371 vs 2685). This may have influenced the results. However, it has also be shown that hypertension, tobacco smoking and alcohol consumption were more frequent in rural than urban [11,13,14]. This is in agreement that non-communicable diseases in general, diabetes in particular should no longer be considered as diseases of the “*affluent, urban or the west*” [15]*.* Other studies also reported similar findings that prevalence of diabetes was similar in men and women and impaired fasting blood glucose was higher in men than women [16]. Promotion of healthy lifestyles and community awareness on diabetes should therefore target both men and women, urban and rural population.

In Malawi, complications of diabetes are common, particularly micro-vascular with prevalence of nephropathy, retinopathy and neuropathy of 34.7%, 34.7% and 46.4% respectively and control of glycemia and hypertension are poor [17]. However, there are well structured and utilised community outreach clinic programmes. These could be made more comprehensive by adding diabetes screening, treatment and follow-up to the package of services being offered. Guidelines from WHO package for essential non-communicable disease (WHO-PEN) could be used in implementing diabetes screening, treatment and follow up programme. The WHO PEN primary health care approach has been recommended as one of cost-effective approaches for delivering interventions for chronic non-communicable diseases including diabetes in resource-poor settings [18-20].

### Limitations of the study

Over-representation of females (70.2%) was one of the limitations of this study. However, it was unlikely that this had an influence on the results because data were weighted (standardised) for age and sex to national population. The over representation of females was not by study design because at household level, eligible participants were randomly selected using the Kish sampling method built-in the PDAs. Refusals/non-availability (though relatively small, 245 (5%) out of 5,451 eligible participants), was another limitation of this study. Specifically, males aged 25–34 years were the ones that were under-represented based on 2008 National Statistical Office population figures (42.5% vs 47.5%, *p* < 0.05). The under representation of men in this age group was due to some being away from home at the time of the survey. It was not known if this group had different survey characteristics. All the other age groups were representative of the national population. There were no differences in the refusal/non-availability between males and females and no replacements were made. About 88% of all the 3,056 participants that were tested for fasting blood glucose were from rural areas. This was not due selection bias but was in line with the population distribution in Malawi which is 85% rural [21].

The other limitation was that the Accutrend *Plus* machines (Roche, Mannheim, Germany) used this study for blood glucose testing were not standardized, quality controlled and capillary was not compared with venous plasma blood tests. However, the machines were calibrated every day using the glucose control strips as per instructions on the use of machines. Diagnosis of diabetes was based on fasting glucose test only. Without oral glucose tolerance test (OGTT) results, the estimated prevalence of 5.6% could be lower than the real prevalence.

Not all participants who consented and adhered to fasting were tested for blood glucose (number consented and adhered to fasting 5023, number tested 3056 (60.8%)). Technical factory fault of the biochemistry machines (Accutrend plus®) led to about 40% of the participants who gave their informed consent and adhered to fasting advice not to be tested. Two machines were sent back to the manufacturer (Roche). It was uncertain whether the failure to measure fasting blood glucose in all the participants who consented had an influence on the results. The curtailment of testing affected men, women and all age groups equally. Based on number of participants and their ages, the available results for each measurement were standardised for age and sex to the national population. The findings presented in this paper were therefore age-sex adjusted estimates. Nevertheless this population based study captured fasting blood glucose tests for over 3000 adults.

## Conclusion

This study has presented evidence on high burden of diabetes mellitus, impaired fasting blood glucose and their risk factors in Malawi. Promotion of healthy lifestyles, community awareness, early detection and treatment could prevent and control this public health problem.

## Competing interests

The authors declare that they have no competing interests.

## Author contributions

KPM, CM and DK conceived and designed the study. KPM, CM and DK conducted the study. KPM analyzed the data: KPM, CM and DK wrote the manuscript: All the authors read and approved the final manuscript.

## Pre-publication history

The pre-publication history for this paper can be accessed here:

http://www.biomedcentral.com/1472-6823/14/41/prepub
